# Association among cardiopulmonary and metabolic rehabilitation, arrhythmias, and myocardial ischemia responses of patients with HFpEF or HFmrEF

**DOI:** 10.1590/1414-431X2024e13174

**Published:** 2024-03-04

**Authors:** C.A.C. Hossri, F.C. Araujo, B.G. Baldi, R. Otterstetter, V.R. Uemoto, C.R.R. Carvalho, L.E. Mastrocola, A.L.P. Albuquerque

**Affiliations:** 1 Hospital do Coração, Associação Beneficente Síria, São Paulo, SP, Brasil; 2 Pneumologia-Incor, Instituto do Coração, Hospital das Clínicas (HCFMUSP), Faculdade de Medicina, Universidade de São Paulo, São Paulo, SP, Brasil; 3 School of Exercise and Nutrition Sciences, University of Akron, Akron, OH, USA; 4 Setor da Bioengenharia, Instituto Dante Pazzanese, São Paulo, SP, Brasil; 5 FCA Sports, Belo Horizonte, MG, Brasil

**Keywords:** Cardiopulmonary rehabilitation, Exercise test, Ischemic heart disease, Arrhythmia, SF-36 quality of life

## Abstract

There's limited evidence of the potential benefits of cardiopulmonary and metabolic rehabilitation (CPMR) in patients with heart failure with preserved ejection fraction (HFpEF) or mildly reduced ejection fraction (HFmrEF) and coronary artery disease (CAD). The aim of this study was to investigate the impact of CPMR on the myocardial ischemia response (MIR), exercise-induced arrhythmias (EIA), New York Heart Association (NYHA) functional class, heart rate recovery (HRR), Borg CR10 perceived symptoms, and the SF-36 physical and mental health summary scores. A prospective cohort study was conducted with 106 patients undergoing 12 weeks of CPMR who completed two exercise tests pre- and post-CPMR: 1) maximum incremental test (CPX) and 2) submaximal constant load test (SUB). After CPMR, the effects on MIR, EIA, NYHA functional class, and HRR during both tests were analyzed. There was a significant change in NYHA functional classes after CPMR, with 96% of the patients in class I (*vs* 62% pre-CPMR, P<0.0001), 4% in class II (*vs* 32%), and none in class III (*vs* 6%). There was a significant reduction in the frequency of EIA (P<0.05) and MIR (P<0.001) and a significantly improved performance on both CPX and SUB tests (P<0.0001). Lastly, there was significant progress in the recovery metrics like HRR (P<0.0001), the Borg CR10 (P<0.0001), and the SF-36 summary scores (P<0.0001). The CPMR resulted in a significant decrease in EIA, delayed ischemia threshold in CPX and SUB tests, increased functional capacity, and improved quality of life.

## Introduction

According to the World Health Organization (WHO), 50% of deaths and complications resulting from cardiovascular diseases (CVD) could be avoided by implementing low-cost global and individual changes to reduce the risk factors (1,2). Through exercise training, cardiopulmonary and metabolic rehabilitation (CPMR) has primary and secondary actions against CVD, improving the patient's quality of life, especially patients with ischemic heart disease (3-6).

The WHO has defined cardiac rehabilitation as: ‘The sum of activities required to influence favourably the underlying cause of the disease, as well as to provide the best possible physical, mental and social conditions, so that the patients may, by their own efforts, preserve or resume when lost as normal a place as possible in the community' (7). Despite consistent international clinical guideline recommendations (3,8), evidence-based CPMR in patients with heart failure (HF) is administered to less than 20% of patients across Europe and the USA (9).

Most of the evidence for cardiac rehabilitation has been reported in patients with heart failure with reduced ejection fraction (HFrEF). Nonetheless, growing literature demonstrates the potential benefits of CPMR in heart failure with preserved ejection fraction (HFpEF) or mildly reduced ejection fraction (HFmrEF) (10).

Therefore, this study aimed to investigate the effects of CPMR on patients diagnosed with HFpEF or HFmrEF and concomitant coronary artery disease (CAD). We evaluated clinical parameters, including the NYHA functional class, heart rate recovery (HRR), Borg CR10 scale, and SF-36 physical and mental health summary scores. Finally, we assessed the effects of CPMR on myocardial ischemia responses (MIR) and the occurrence of exercise-induced ventricular or atrial arrhythmias (EIA).

## Material and Methods

### Study population

One hundred and six patients with HFpEF or HFmrEF and CAD were recruited continuously throughout this study; informed consent was obtained from each patient. This research was performed at the Department of Cardiac, Pulmonary and Metabolic Rehabilitation of the Heart Hospital (HCor) of São Paulo. The study protocol complied with the ethical guidelines of the 1975 Declaration of Helsinki and was approved by the Ethics Committees in Research of the Heart Hospital (CEP/HCor) and the University of São Paulo Medical School (#048/11).

### Participation criteria and exercise tests

Patients were enrolled in the study from April 2011 to March 2013. The inclusion criteria were: 1) patients diagnosed with ischemic heart disease, where coronary artery disease (CAD) was established based on either a clinical history of angina or laboratory confirmation through coronary angiography; 2) myocardial infarction (occurring more than one month before inclusion); 3) patients who underwent surgical myocardial revascularization or percutaneous coronary intervention (PCI), with or without stent implantation (at least one week before enrollment); and 4) any sex and age, provided they were over 18 years old. The exclusion criteria were: 1) patients with a left ventricular ejection fraction less than 35% as determined by Simpson's method; 2) individuals classified as NYHA functional class IV; 3) those with musculoskeletal disorders or any other medical condition preventing them from exercising; 4) patients suffering from severe pulmonary diseases necessitating oxygen supplementation; 5) individuals in advanced stages of malignancies and those with obesity with a body mass index (BMI) higher than 35 kg/m^2^; and 6) participants who failed to complete a minimum of 50% of the prescribed physical training sessions or those who did not undergo all four cardiorespiratory tests in accordance with the research protocol. After signing an informed consent form, patients performed an incremental maximal exercise test (CPX) (11) and later a submaximal constant load (SUB) (12) at 80% of the maximum load achieved during the CPX test. The same CPX and SUB tests were repeated after completion of the CPMR. All tests were performed on a treadmill (Super ATL; Inbramed, Brazil) with direct breath-by-breath gas exchange measurements (Medgraphics; Ultima CardiO2; Breeze software suite v6.4, USA). The reference values used for VO_2_ and ventilatory thresholds (VT) followed the normative data by Hansen et al. (13). VT1 was determined by the V-slope method (14) and VT2 was determined by the respiratory compensation point method (15). In addition, all exercise tests (ETs) were terminated by the following criteria: exhaustion, significant dyspnea, progressive angina, complex arrhythmias (supraventricular tachycardia, sustained ventricular tachycardia), and exercise variables according to the guidelines of the American Heart Association (3) and the Brazilian Society of Cardiology (16).

### The cardiopulmonary and metabolic rehabilitation program

The CPMR comprised supervised exercise with aerobic and resistance sessions, a full-body warm-up, and static flexibility exercises. Patients trained thrice weekly for approximately 60 min and remained monitored by a multichannel telemetry system (Quinton Qtel RMS; Welch Allyn, USA). The rehabilitation sessions started with 40 min of indoor (treadmill) exercise. The first 4 sessions were completed at a steady-state intensity and moderate intensity between VT1 and VT2. The upper limit was determined during the first CPX test by the occurrence of chest pain, ECG ST depression, or arrhythmias. During the rehabilitation sessions, monitored patients remained 10 bpm below the threshold determined in the CPX test. After the aerobic training, patients completed resistance training 2-3 times per week, 20 min (duration) sessions, with 3 sets of 10 reps at 40-50% of maximal voluntary strength and 1 min rest in between. They alternated two lower body sessions with one upper body session. Maximal voluntary contraction (MVC) was estimated by correlation with the weight needed for sets of 15 reps ending at Borg RPE 13 (mild to moderate fatigue) (17). The aerobic training intensity was set based on the treadmill speed (km/h) and the patient's heart rate (beats per minute) according to the VT1 as determined during the first CPX test (18). The upper limit of the aerobic training also considered the occurrence of the ST-segment depression >1.0 mm, with a horizontal or descending shape, clinical signs of chest pain, initiation of complex ventricular or atrial arrhythmias, or tachyarrhythmia events. After the first four sessions of moderate constant aerobic training, moderate to high-intensity interval training (HIIT) was added to the CPMR program to improve patients' adherence and avoid training monotony. The HIIT sessions started with a 10-15 min warmup at 50-70% VO_2_peak, followed by 2×3 min at 80-90%, 4 min of recovery at 60-70%, and 10-15 min of cool-down at 50-70%. The intensity was based on heart rate ranges determined during the first CPX test (19).

### Clinical and electrocardiographic analysis

In addition to the clinical assessments related to the NYHA functional class, ischemic and arrhythmic responses were analyzed by electrocardiographic (ECG) tracings obtained in twelve simultaneous leads (Quinton Q-Stress; Welch Allyn). The criteria used to assess MIR on the ECG involved visually analyzing the ST-segment, specifically looking for the J point depression (≥1.5 mm). The measurement for the ascending ST-segment was taken 80 ms after the J point. Furthermore, these had to be present in at least four successive beats (stable baseline) in two or more classic ECG derivations. Moreover, if the ST-segment morphology was horizontal or descending, the 1.0 mm drop in level was measured at the J point as a positivity criterion according to the Brazilian Society of Cardiology guidelines on exercise testing (16).

The clinical criteria adopted for MIR were retrosternal pain or discomfort suggestive of coronary insufficiency. Additional features included an oppressive, progressive, and reproducible feeling during the ETs. Arrhythmias were counted as they occurred during the test, and the arrhythmogenic density was calculated as the ratio of extrasystoles to test duration. The test duration included the 2 min before the test, the test duration itself, and the 6 min of the recovery phase. EIAs were considered for analysis when: 1) there were isolated ventricular or supraventricular ectopic beats greater than six events per test or above 1% of the total test heartbeats; and 2) there was repetitive supraventricular or ventricular tachycardia during the ETs. All arrhythmogenic events and MIRs were analyzed in the ETs before and after the rehabilitation program. HRR was also evaluated in the 1st min of the recovery period.

### Exercise perceived symptoms and quality of life

Borg CR10 scale measurements for dyspnea and leg discomfort were recorded at the end of all ETs (20). The patient's quality of life before and after the CPMR was surveyed with the Brazilian-Portuguese version of the SF-36 health-related instrument. Two registered psychologists applied the questionnaires and compiled the results. Although the questionnaire's scoring is objective, the process was not blinded. Results are reported as physical and mental health summary scores (21-23).

### Statistical analysis

Data are reported as means±SD for variables with normal distribution, median (Mdn) and the interquartile range (IQR 25th-75th range) for non-normal distribution, or as numbers (percentages). The Shapiro-Wilk test was used to determine the variables' normality condition. A paired *t*-test was used to compare variables with normal distribution, whereas the Wilcoxon test was used to compare those with non-normal distribution. Categorical variables are reported as percentages and were compared with the nominal symmetry test. Pearson and Spearman correlation coefficients were used to evaluate the association between variables. Group differences were considered significant if the P<0.05. Data were analyzed with RStudio v2022.07.2 build 576 (Posit Software, USA).

## Results

Of the 106 patients screened for this study, 43 were three-vessel, 27 were two-vessel, and 24 were single-vessel CAD. Twelve patients with no coronary angiography had a positive clinical history and an electrocardiographic and/or echocardiographic exam for an ischemic event. Severe valvular diseases were not included, and there were no important valvular disorders in the sample. Thirty-seven patients were excluded: 31 for not following the rehabilitation program, 3 because of a BMI over 35 kg/m^2^, and 3 for having a mean left ventricular ejection fraction (LVEF) lower than 35%.

Sixty-nine patients completed the protocol and had 83.4% session compliance. They were mostly males, with CAD as the primary etiology and systemic arterial hypertension as the most common comorbidity. Most patients were taking statins, aspirin, and beta-blockers, with a high prevalence (88%) of patients using two or more classes of medications. Most of the patients (78%) had invasive coronary treatment (surgery or percutaneous) eight months or more before starting CPMR (Table 1).

**Table 1:** Clinical characteristics of the patients before cardiopulmonary and metabolic rehabilitation.

Age (years)	60.5±11.5
Gender (male/female, %)	59 (85.3%)/10 (14.7%)
Mean LVEF (n=61), %	55.7±10.1
CPMR compliance, %	83.4±10.3
Drugs	
Statins	63 (91.3%)
Aspirin	64 (92.8%)
Beta-blockers	60 (87.0%)
Diuretics	32 (46.4%)
ACEI/ARB	57 (82.6%)
Comorbidities	
Overweight (BMI: 25.1-30.0)	33 (47.8%)
Obesity (BMI: 30.1-35.0)	18 (26.4%)
Systemic arterial hypertension	56 (81.1%)
Diabetes	28 (41.1%)
Dyslipidemia	49 (71.0%)
Smoking	
Current	7 (10.3%)
Previous	42 (61.7%)
Never	20 (28%)
CAD clinical history	
Myocardial infarction	41 (59.4%)
CABG	26 (37.6%)
PCI without MI	32 (46.3%)
MI+CABG	14 (14.7%)
MI+CABG+PCI	7 (10.2%)
CAD treatment	
Clinical only	15 (22.0%)
Invasive (PCI or CABG)	54 (78.0%)

Data are reported as percent or means±SD. BMI: body mass index (kg/m^2^); ACEI: angiotensin-converting enzyme inhibitors; ARB: angiotensin II receptor blockers; CABG: coronary artery bypass grafting; CAD: coronary artery disease; LVEF: left ventricular ejection fraction; MI: myocardial infarction; PCI: percutaneous coronary intervention.

### Clinical and electrocardiographic metrics

**Figure 1:**
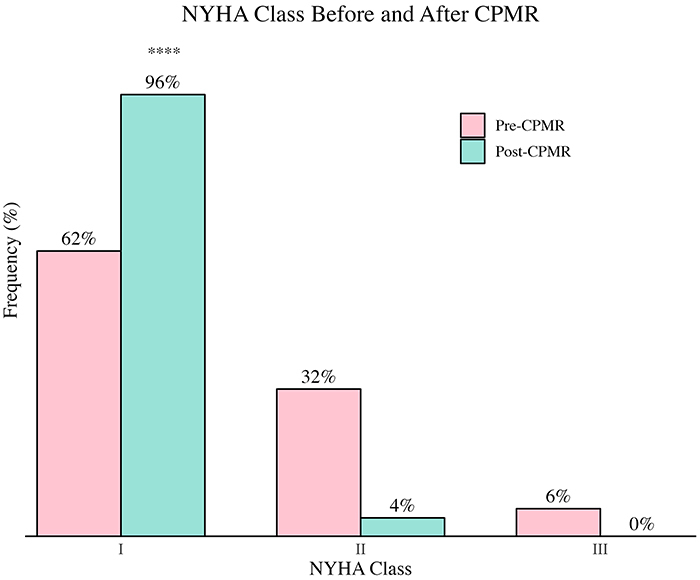
Change in New York Heart Association (NYHA) functional class pre- and post-cardiopulmonary and metabolic rehabilitation (CPMR). Data are reported as percentages (n=69). ****P<0.0001 for change from class II to I after CPMR (nominal symmetry test).

Pre-CPMR NYHA functional classes were predominantly I and II, and after CPMR, the status of most patients changed to class I (95.6%), while no one was classified as class III or IV (Figure 1). All included patients exhibited a mean LVEF above 35%. However, among the 61 patients with both pre- and post-CPMR echocardiographic assessments, 10 had LVEF between 40 and 50% and 51 patients had LVEF of 50% or higher; 8 patients did not have pre- and post-CPMR echocardiographic studies available. Patients with a mild reduction in LVEF had significantly increased resting LVEF (55.7±10.1 *vs* 58.2±10.3, P<0.001), although not clinically relevant.

**Figure 2:**
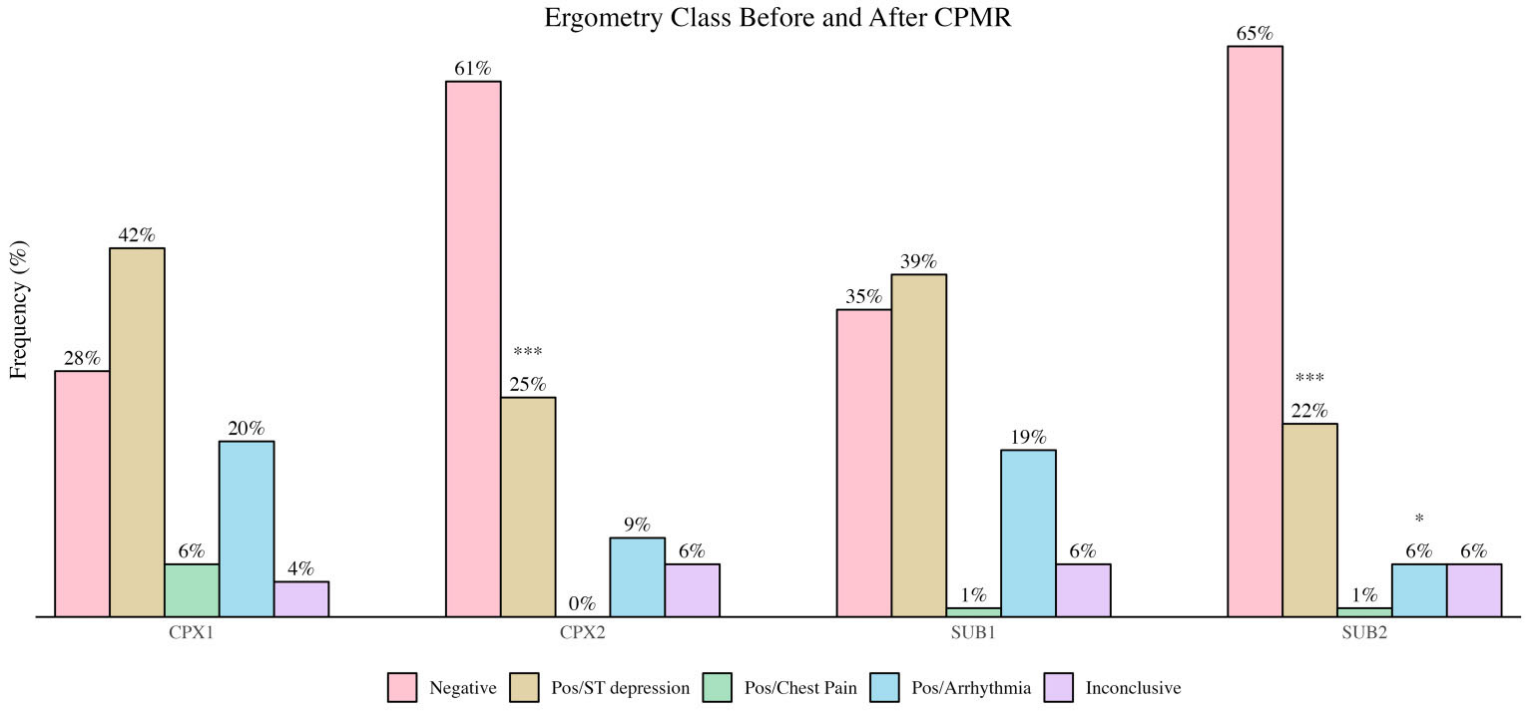
Maximum incremental test (CPX) and submaximal constant load test (SUB) changes in myocardial ischemia response and exercise-induced arrhythmias through exercise tests before (1) and after (2) cardiopulmonary and metabolic rehabilitation (CPMR). Data are reported as percentages (n=69). Pos: positive test with ST depression, chest pain, or arrhythmia. *P<0.05 for change from SUB1 Pos/Arrhythmia to SUB2 Negative. ***P<0.001 for change from CPX1/SUB1 Pos/ST depression to CPX2/SUB2 Negative (nominal symmetry test).

Before CPMR, there was a high percentage of patients with ST-segment depression of ≥1.0 mm and complex (supraventricular or ventricular) arrhythmias in the ETs. In the ETs, ECG changes and clinical manifestations showed a significant reduction in positivity for MIR. That is, the ECG ST-segment deviations were delayed or abolished. A comparison of the trials (electrocardiographic, clinical, or ischemic arrhythmia manifestations) pre- and post-CPMR showed a significant reduction in positive CPX (68 *vs* 34%, P<0.001) and SUB (59 *vs* 29%, P<0.001) tests (Figure 2).

### Physical performance and cardiovascular metrics

**Figure 3:**
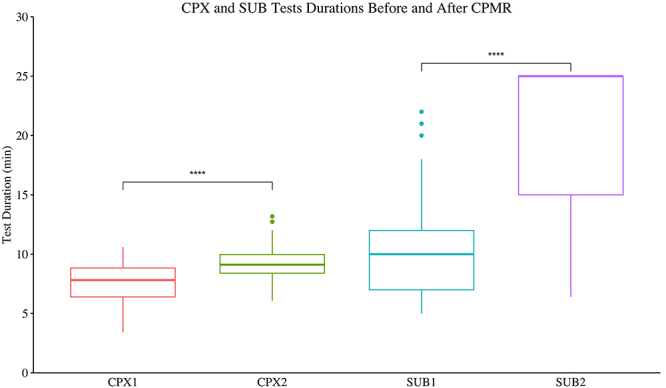
Maximum incremental test (CPX) and submaximal constant load test (SUB) durations before (1) and after (2) cardiopulmonary and metabolic rehabilitation (CPMR). Data are reported as median and the interquartile range (25th-75th); n=68. ****P<0.0001, Wilcoxon test. HRR: Heart rate recovery (drop).

**Figure 4:**
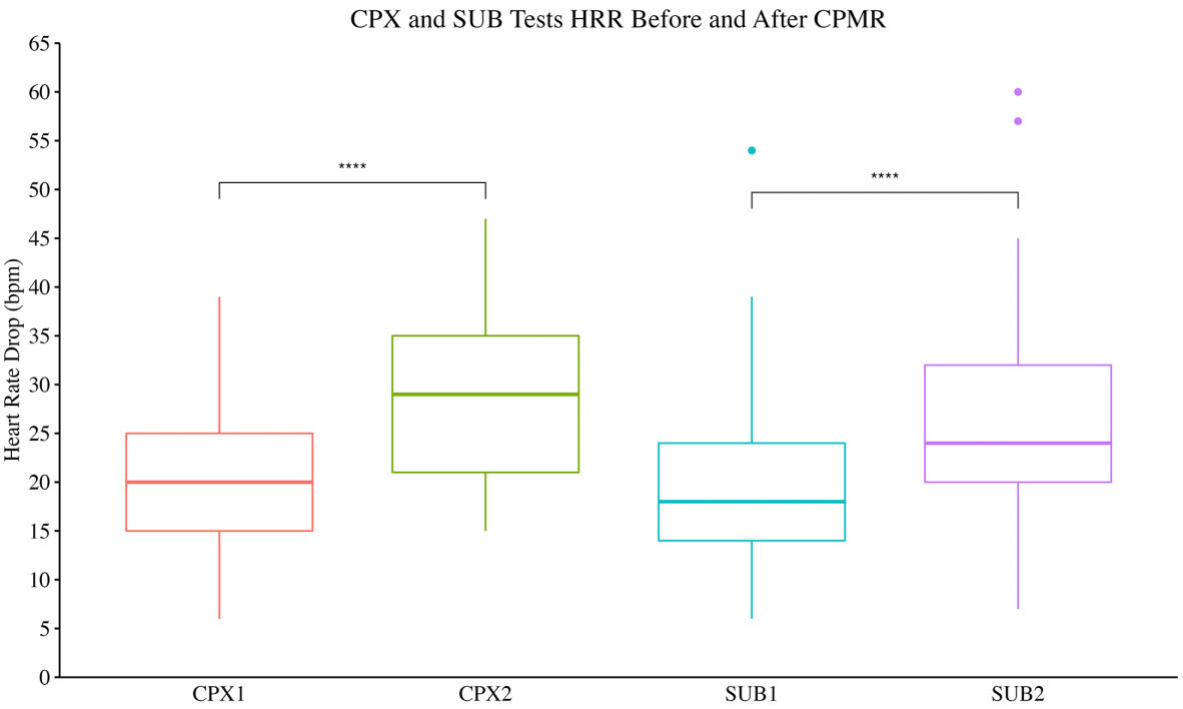
Maximum incremental test (CPX) and submaximal constant load test (SUB) heart rate recovery (drop) (HRR) in the 1st min after exercise tests before (1) and after (2) cardiopulmonary and metabolic rehabilitation (CPMR). Data are reported as median and the interquartile range (25th-75th); n=69. ****P<0.0001, Wilcoxon test.

Patients attained higher exercise performances after CPMR, with higher treadmill speeds and total distance covered (Table 2). In the SUB test, patients almost tripled the exercise performance represented by longer test durations (Mdn 10 min (IQR=7-12) *vs* 25 (15-25), P<0.0001). The SUB was more sensitive in detecting improvement in exercise capacity than CPX (Figure 3). HRR in the 1st min of the recovery phase in the exercise tests was also significantly boosted after the CPMR in the CPX (Mdn 20 bpm (IQR=15-21) *vs* 29 (21-35), P<0.0001) and SUB (18 bpm (14-24) *vs* 24 (20-32), P<0.0001) tests (Figure 4).

**Table 2:** Maximum incremental test metrics pre- and post-cardiopulmonary and metabolic rehabilitation (CPMR).

	Pre-CPMR	Post-CPMR	P
Duration (min)	7.59±1.53	9.49±2.41	0.001
Distance (m)	659.5±222.4	893.4±268.1	<0.001
Peak treadmill speed (km/h)	5.6±1.7	6.7±1.5	<0.0001
Peak treadmill grade (%)	2.2±2.8	3.5±3.0	0.029

Data are reported as means±SD (*t*-test).

### Exercise perceived symptoms and quality of life

Despite the longer exercise durations, the Borg CR10 measurements for dyspnea [CPX Mdn 8.0 (IQR=7-9) *vs* 5.0 (5-7), P<0.0001 and SUB 7.0 (6-7) *vs* 5.0 (4-6), P<0.0001] and leg discomfort [CPX 7.0 (7-8) *vs* 6.0 (5-7), P<0.0001 and SUB 7.0 (7-8) *vs* 5.0 (4-6), P<0.0001] were significantly reduced after CPMR.

The patient's quality of life measured by the SF-36 physical [Mdn 67.8 (IQR=55-82) *vs* 83.5 (76-91), P<0.0001] and mental health [67.8 (54-83) *vs* 83.0 (75-92), P<0.0001] summary scores were improved significantly following CPMR.

## Discussion

Most scientific evidence for CPMR has been reported in patients with HFrEF. Despite the fear of acute and severe cardiovascular events during CPMR sessions, several mechanisms are associated with exercise-induced cardioprotection, such as changes to the heat shock proteins and the nitric oxide pathway, increased antioxidant capacity, improvement in the function of ATP-dependent potassium channels, and activation of the opioid system (24,25). These mechanisms uncover the health benefits observed in many studies and reinforce the importance of CPMR for patients with HF and CVD. However, there is insufficient data to fully determine the impact of CPMR on clinical and cardiovascular variables, including the NYHA functional class, MIR, HRR (26), and cardiac arrhythmias in patients with HFpEF or HFmrEF and CAD (9,19,27,28).

ETs are routinely used to assess a patient's functional capacity and prognosis. Several physiological markers are monitored and analyzed during and after ETs. Maximum oxygen consumption (VO_2_ max), energy expenditure in metabolic equivalents (MET), chronotropic response (CR), and HRR are some examples that have a proven prognostic value (4,5,19). Such tests are typically carried out on a treadmill and apply incremental or stepped ramp protocols until exhaustion. The Bruce et al. (29) and Ellestad et al. (30) protocols are the most used in the diagnostic and prognostic evaluation of CVD and the prescription of physical training. However, many patients may not reach their maximum work capacity, and without accurate maximum measurements, the CPX tests may not show the expected physiological changes after CPMR. Therefore, submaximal constant load tests may better assess the magnitude of the CPMR effects (31).

Larsen et al. observed that submaximal tests at constant load have good sensitivity in evaluating the multifactorial physiological changes after training. However, their investigation examined the differences between a 6-min walk and maximal exercise tests on a cycle ergometer on patients with congestive heart failure (NYHA class III, mean ejection fraction of 32.5%) (31). Considering that the SUB tests correspond more closely to daily life activities than the CPX tests, our results indicated that the SUB tests were superior in quantifying the physiological changes after CPMR.

Our CPMR program had high levels of patient supervision and session adherence. Despite the mild limitation in terms of baseline NYHA functional class (I-II in 94% of patients), all had CAD (60% with previous myocardial infarction) and associated comorbidities. After training, they had significant improvements in the cardiovascular variables. The ischemic alterations on the ECG and complex arrhythmias decreased significantly in both CPX and SUB tests.

Regular exercise increases the coronary blood flow in CAD patients, delaying or eliminating the ischemic threshold (32). Such changes could explain the improvement in the manifestations of myocardial ischemia by improving the anginal symptoms and lowering the magnitude of ST-segment depression. However, Zdrenghea et al. (33) showed that exercise-induced ST-segment depression was markedly attenuated in high-risk patients during consecutive exercise sessions. Similarly, Lambiase et al. (34) trained patients with CAD before PCI and observed that ST-segment deflation, expected in PCI, decreased in these patients. These results indicate the promising use of exercise in promoting clinical cardioprotection.

The main mechanisms of cardioprotection by exercise training include an increase in the heat shock protein (HSP) production, involvement of the nitric oxide (NO) pathway, an increase in the cardiac antioxidant capacity, a functional improvement in the ATP-sensitive potassium (KATP) channels, and activation of the opioid system (35). For arrhythmias, it has already been shown that exercise training can decrease sympathetic activity in healthy subjects and heart failure (36). *In vivo* studies have demonstrated that catecholaminergic polymorphic ventricular tachycardia in mice had a good tolerance and benefited from exercise training (37). Thus, exercise training in the subacute to chronic phase of the MI did not increase the risk of malignant arrhythmias but restored autonomic function and cardiac electrical stability (38).

Cohen-Solal et al. (39) have reported that prolonged oxygen consumption recovery appears partly related to the slow recovery of energy stores in the peripheral skeletal muscles. It has been reproducible and largely independent of whether the test was maximal or submaximal. In our study, we observed a significant improvement in heart rate recovery in the 1st min after the CPMR. The CPMR also significantly impacted exercise tolerance, with lower Borg CR10 scores at the end of the tests and higher SF-36 physical and mental health summary marks. Improvement in the autonomic nervous system modulation through reduced sympathetic tonus and central and peripheral cardiovascular and metabolic adaptations may have led to increased oxygen delivery during and after exercise.

### Limitations

This study had a prospective cohort design without a control group, as all participants were tested before and after being exposed to CPMR. The recruitment process was not randomized or blinded. It aimed to study the responses from patients with ischemic heart disease who underwent different exercise tests and a comprehensive cardiopulmonary rehabilitation program. Attrition bias may have influenced our study results as 35% of the recruited patients had not completed more than 50% of the CPMR sessions and were excluded. Finally, the reporting of exercise tests and quality of life questionnaire scoring were not blinded.

### Conclusions

The findings of this rehabilitation study underscore the transformative impact of exercise training on cardiovascular health, physical performance, and overall well-being among patients with a history of CAD. It highlighted how a targeted intervention can optimize patient outcomes by improving their NYHA functional capacity and delaying the ischemia threshold or the occurrence of EIA during moderate to high-intensity exercise. Finally, our results warrant future investigations into the nuanced impacts of rehabilitation strategies for distinct patient CAD profiles.
